# Role of Dietary Fiber and Energy Intake on Gut Microbiome in Vegans, Vegetarians, and Flexitarians in Comparison to Omnivores—Insights from the Nutritional Evaluation (NuEva) Study

**DOI:** 10.3390/nu15081914

**Published:** 2023-04-15

**Authors:** Waldemar Seel, Sarah Reiners, Kristin Kipp, Marie-Christin Simon, Christine Dawczynski

**Affiliations:** 1 Nutrition and Microbiota, Institute of Nutrition and Food Sciences, University of Bonn, 53012 Bonn, Germany; 2 Junior Research Group Nutritional Concepts, Institute of Nutrition, Friedrich Schiller University Jena, 07743 Jena, Germany; 3 Department of Pediatrics and Adolescent Medicine, Sophien- and Hufeland Hospital, Henry-van-de-Velde-Str. 1, 99425 Weimar, Germany

**Keywords:** western diet, flexitarians, vegetarians, vegans, energy intake, dietary fibers, fecal microbiome composition

## Abstract

In recent years, there has been a global trend towards a plant-based lifestyle. In the NuEva study, dietary self-reports of 258 participants following one of four diets (Western diet (WD), flexitarians (Flex), vegetarians (VG), and vegans (VN)) were related to fecal microbiome composition. Reduced consumption of animal products (VN < VG < Flex < WD) was associated with a decreased intake of energy (*p* < 0.05), and an increased intake of soluble and non-soluble dietary fibers (*p* < 0.05). We observed the lowest average microbiome diversity in vegans and the highest in WD. Compared to WD, VG (*p* < 0.05) and VN (*p* < 0.01) differed significantly in their bacterial composition. These data were related to dietary fiber intake. Furthermore, we identified 14 diet-specific biomarkers at the genus level by using LefSe analysis. Of these, 11 showed minimum or maximum counts in WD or VN. While the VN-specific species were inversely associated with cardiovascular risk factors, a positive association was detected for the WD-specific species. Identifying biomarkers for the diets on extreme ends of the spectrum (WD and VN) and their association with cardiovascular risk factors provides a solid evidence base highlighting the potential and the need for the development of personalized recommendations dependent on dietary patterns. Even so, the mechanisms underlying these diet-specific differences in microbiome composition cannot yet be clearly assessed. The elucidation of these associations will provide the basis for personalized nutritional recommendations based on the microbiome.

## 1. Introduction

There is a global rise in vegetarianism and veganism, with an estimated one billion people worldwide adopting a vegetarian-vegan lifestyle [1]. This lifestyle is characterized by omitting defined food groups such as meat, sausage, fish (vegetarians (VG)), or additionally, dairy products and honey (vegans (VN)). This bears the risk of undersupply of valuable nutrients. Unfavorable aspects of the vegetarian-vegan lifestyle are inadequate intakes of B vitamins, vitamin D, long-chain omega-3 fatty acids, calcium, iron, iodine, selenium, and zinc. In addition, a vegetarian/vegan diet, usually rich in fruits, vegetables, whole-grains, legumes, nuts, and various soy products, is also characterized by a lower intake of energy, saturated fat, and cholesterol and a higher intake of dietary fiber, carotenoids, vitamins, and health-promoting phytochemicals [2,3,4]. The Western diet (WD) is the primary nutritional style in Germany [5]. The traditional WD is characterized by high intake of energy, saturated fat, salt, and simple or added sugar, and a comparably low intake of vegetables and fruits resulting in low intake of dietary fibers, polyunsaturated fatty acids, and secondary plant compounds [6]. The daily energy intake, as observed during the screening period of the NuEva study increased in the following order: VN < VG < flexitarians (Flex) < WD. The consumption of soluble and insoluble dietary fibers was the highest in VG and markedly lower in omnivores [2].

The human gut microbiota is dominated by two Bacteroidetes phyla (which includes Bacteroides and Prevotella) and Firmicutes (which includes Clostridium, Enterococcus, Lactobacillus, and Fecalibacterium), while Actinobacteria (mainly Bifidobacterium), Proteobacteria, Verrucomicrobia, and Archaea play a minor part [7]. Evidence from several available observational studies describe clear differences in the microbiota composition between omnivores, vegetarians, and vegans [8]. This indicates that nutrient intake influences the composition of the human gut microbiome, which in turns influences numerous functions in the human body, such as nutrient metabolism and immune defense [9]. In this context, the gut microbiota plays an important role in the fermentation of dietary fibers resulting in the production of short-chain fatty acids (SCFAs), which are important signaling molecules involved in the regulation of metabolism and inflammation [10]. In this paper, we explore the relationship between varying intakes of dietary fibers and energy in the examined diets and the composition of the gut microbiome. From these data, personalized dietary recommendations based on dietary patterns and focusing on energy and fiber intake can be made.

## 2. Materials and Methods

### 2.1. Study Design

The NuEva study was a parallel-designed trial with participants following one of four diets (WD, Flex, VG, VN). The adherence to one of these diets for at least 1 year before enrollment was a precondition and was proved by lifestyle questionnaires and a food protocol.

**Figure 1:**
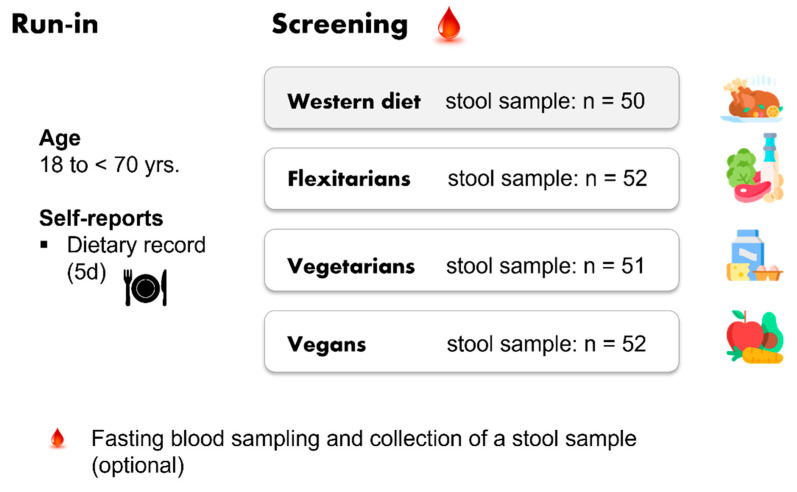
Study design—NuEva screening.

The participants were recruited in central Germany (recruiting area: Jena-Halle-Leipzig) from spring to summer 2018 according to defined inclusion and exclusion criteria [11]. The allocation ratio was approx. 1:1:1:1 and the long-term trial over 24 months consisted of four study periods (run in, screening, intervention, and follow up) [11]. The run-in period of NuEva study utilized self-reporting of individual dietary habits that included prepared protocols to record and document the variety in dietary practices within each group (report period: 5 d). In addition, questionnaires to assess long-term nutritional habits, socio-economic status, physical activity, health and disease status (including medication use) were used. In the screening period, blood samples were taken and 24 h urine was collected to establish nutrient status and selected risk factors for cardiovascular diseases (CVD), type-2 diabetes mellitus, and inflammatory markers (Figure 1). In addition, health checks were carried out and included measurements of weight, height, waist circumference, blood pressure, and bioelectrical impedance analysis (BIA). Optionally, a stool sample was collected at the beginning of the NuEva study (Figure 1) [11].

The herein presented data are from the screening from the NuEva study. The protocol and further investigations of the NuEva study are described elsewhere [2,11].

The study protocol was reviewed and approved by the Ethical Committee of the Friedrich-Schiller-University of Jena (number: 5504-03/18). The NuEva study was registered before launching (Clinical-Trials.gov Identifier: NCT03582020).

### 2.2. Microbiome Analysis

#### 2.2.1. DNA Extraction

Stool samples were collected from the participants and frozen at −80 °C immediately after delivery. Sample transfer was performed on dry ice. Total genomic DNA was extracted from 120 mg fecal material using the ZR BashingBead lysis tubes (0.1 and 0.5 mm, Zymo Research, Freiburg, Germany) in combination with the chemagic DNA Stool Kit (Perkin Elmer, Rodgau, Germany) according to the manufacturer’s instructions [12,13,14]. A mechanical lysis step was added after the addition of the lysis buffer using the Precellys 24 Tissue Homogenizer (Bertin Instruments, Frankfurt am Main, Germany). After extraction, DNA was stored at −20 °C until further analysis.

#### 2.2.2. Library Preparation

Amplicon sequencing of the fecal microbiome was done at the Life & Brain GmbH. Briefly, the V3V4 region of the 16S rRNA gene was amplificated in a first PCR step using the primer Bakt_341F (5′-CCTACGGGNGGCWGCAG-3′) and Bakt_805R (5′-GACTACHVGGGTATCT AATCC-3′) in a 25 µL PCR reaction containing 2.5 µL template (5 ng/µL), 12.5 µL 2× KAPA HiFi HotStart ReadyMix (Roche, Mannheim, Germany), and 5 µL of corresponding primers (1 µM). PCR was conducted in a thermal cycler as follows: initial denaturation step at 95 °C for 3 min followed by 25 cycles of denaturation (30 s at 95 °C), annealing (30 s at 55 °C), elongation (30 s at 72 °C), and a final elongation step at 72 °C for 5 min. In a second PCR step, dual indices and an Illumina sequencing adapter were added using the Nextera XT v2 Index Kit (Illumina, San Diego, CA, USA). For the second PCR reaction, 25 µL 2× KAPA HiFi HotStart ReadyMix (Roche, Mannheim, Germany), 5 µL of corresponding Nextera XT index primer, and 10 µL PCR grade water in a total volume of 50 µL were used per sample. Cycling conditions used were as follows: initial denaturation at 95 °C for 3 min followed by 8 cycles of denaturation (30 s at 95 °C), annealing (30 s at 55 °C), elongation (30 s at 72 °C), and a final elongation step at 72 °C for 5 min. After each PCR step, amplicon libraries were spot-checked on an Agilent TapeStation 4200 using D1000 ScreenTape (Santa Clara, CA, USA) and were purified using AMPure XP beads (Beckman Coulter, Krefeld, Germany). Samples were normalized to 4 nM and pooled equimolar.

#### 2.2.3. 16S rRNA Sequencing

The final pool was quantitated by Qubit dsDNA HS Assay Kit from Thermo Fisher Scientific (Waltham, MA, USA) and fragment size was determined on a D1000 ScreenTape. Sequencing was carried out on a MiSeq system from Illumina using MiSeq Reagent Kit v3 with 2 × 300 cycles. Clustering was conducted at 6 pM with a 15% spike-in of PhiX. Demultiplexing was carried out on the MiSeq system.

#### 2.2.4. Bioinformatics

16S sequencing data were processed using QIIME 2 version 2022.8 [15]. Briefly, sequence quality control and denoising were performed using DADA2 [16]. The quality control step also included the filtering of PhiX reads and chimeric sequences. The sequences obtained after denoising were then classified using SILVA databases to identify amplicon sequencing variants (ASVs) for sequences with >99% sequence similarity. A rarefied table with a sampling depth of 17,500 sequences was used to calculate alpha and beta diversity metrics. A LefSe analysis was performed to identify dietary specific biomarkers. Then, the data were uploaded to the MicrobiomeAnalyst platform for Marker Data Profiling [17,18].

### 2.3. Statistical Analysis

All statistical analyses were performed using GraphPad Prism software version 9.3.1 (GraphPad Software, Inc., San Diego, CA, USA). Baseline characteristics across all four dietary groups were compared using a 1-factor ANOVA. Pairwise significance for WD against Flex, VG, and VN diets was calculated using an independent *t*-test. The linear relationship between the macronutrients and alpha diversity metrics were tested using the Pearson correlation analysis. A pairwise PERMANOVA test was used to identify significant differences of various beta diversity metrics inside QIIME2. The work flow of the statistical assessment is summarized in Supplementary Figure S1.

## 3. Results

### 3.1. Relationship between Different Diets and Alpha Diversity as Well as Beta Diversity Metrics of the Baseline Microbiome

**Figure 2:**
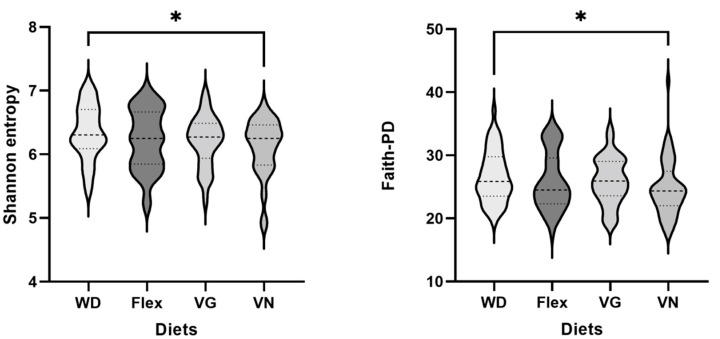
Violin plots showing distribution of alpha diversity metrics Shannon entropy and Faith-PD for each diet. Significant differences only detected for pairwise comparison (unpaired *t*-test) of WD versus VN for both metrics (* *p* < 0.05).

Alpha diversity metrics (Shannon entropy, Pielou’s evenness, Faith-PD) were calculated in Qiime2 and used for subsequent statistical analysis. Based on the ANOVA test, there was no significant difference in the richness of the gut microbiome across all diets tested (Table 1). However, there was a tendency for the vegan group to have the lowest average microbiome diversity and for the WD to have the highest average microbiome diversity (Figure 2). Each diet was tested against the WD, which also doubled as a reference. Table 1 and Figure 2 display the significant differences in bacterial richness between WD and VN, with VN having a lower average diversity than WD.

The data on beta diversity demonstrates significant compositional differences in the gut microbiomes across all diets. This was true for qualitative (Jaccard), quantitative (Bray-Curtis) as well as phylogeny-incorporating measures (Unifrac). In addition, the diets were tested in a pairwise comparison against the WD. No significant differences of the bacterial composition were observed for the Flexitarian group, whereas the dietary patterns VG (*p* < 0.05) and VN (*p* < 0.01) differed significantly in their composition when compared to the WD (Table 2).

### 3.2. Influence of High vs. Low Intake of Dietary Fiber on Gut Microbiota

Since our study focuses on dietary fiber intake, all samples, regardless of their dietary form, were divided into two groups—high fiber intake (>30 g/day) and low fiber intake (<30 g/day). The subdivision, based on the recommendations of the German Society for Nutrition e.V. [19], resulted in approximately equal-sized groups of high and low fiber intake in all diet groups. Whereas the high dietary fiber high group was mainly made up of samples from VN and VG, the low dietary fiber group was made up of samples from the Flex and WD diets. Based on this grouping, no significant differences were found for the alpha diversity metrics Faith-PD (*p* = 0.09) and Shannon entropy (*p* = 0.68). The relatively substantial difference in *p*-values might indicate that possible differences between the fiber content groups could be driven by phylogenetic rather than quantitative differences in microbial richness. The four beta diversity metrics mentioned above also showed significant differences in the fecal bacterial composition between samples with high fiber and low fiber intake (unweighted UniFrac *p* = 0.021; weighted UniFrac *p* = 0.017; Bray-Curtis *p* = 0.001; Jaccard *p* = 0.001), which we had anticipated. The comparison of high and low fiber intake within the diet groups demonstrated a significant difference for Bray-Curtis and Jaccard in VG (*p* = 0.05; Table 3). In addition, we observed a trend for unweighted UniFrac in VG and VN (*p* ≤ 0.1), for weighted UniFrac (*p* ≤ 0.1) in WD and Flex as well as for Bray-Curtis and Jaccard in WD (*p* ≤ 0.1; Table 3).

Furthermore, we confirmed whether significant differences in beta diversity were still visible between diets in comparison to the WD if separated into high and low fiber or if the changes were mainly driven by fiber content consumed (Table 4). Based on all beta diversity metrics, we did not observe significant differences between flexitarians and people of the reference diet, regardless of fiber intake. For vegetarians, significant differences in microbial compositions in comparison to WD were only found inside the low fiber group (weighted UniFrac, *p* = 0.008; Bray-Curtis, *p* = 0.003; Table 4). For vegans, significant differences in microbial compositions in comparison to WD were detected in the high fiber group (unweighted UniFrac, *p* = 0.030; weighted UniFrac, *p* = 0.026; Bray-Curtis, *p* = 0.004; Jaccard, *p* = 0.006) and the low fiber group (weighted UniFrac, *p* = 0.049; Bray-Curtis, *p* = 0.001; Jaccard, *p* = 0.043; Table 4).

### 3.3. Role of Dietary Carbohydrates, Fiber, and Energy Intake on Baseline Gut Microbiome in Dependence of the Diet Group

**Figure 3:**
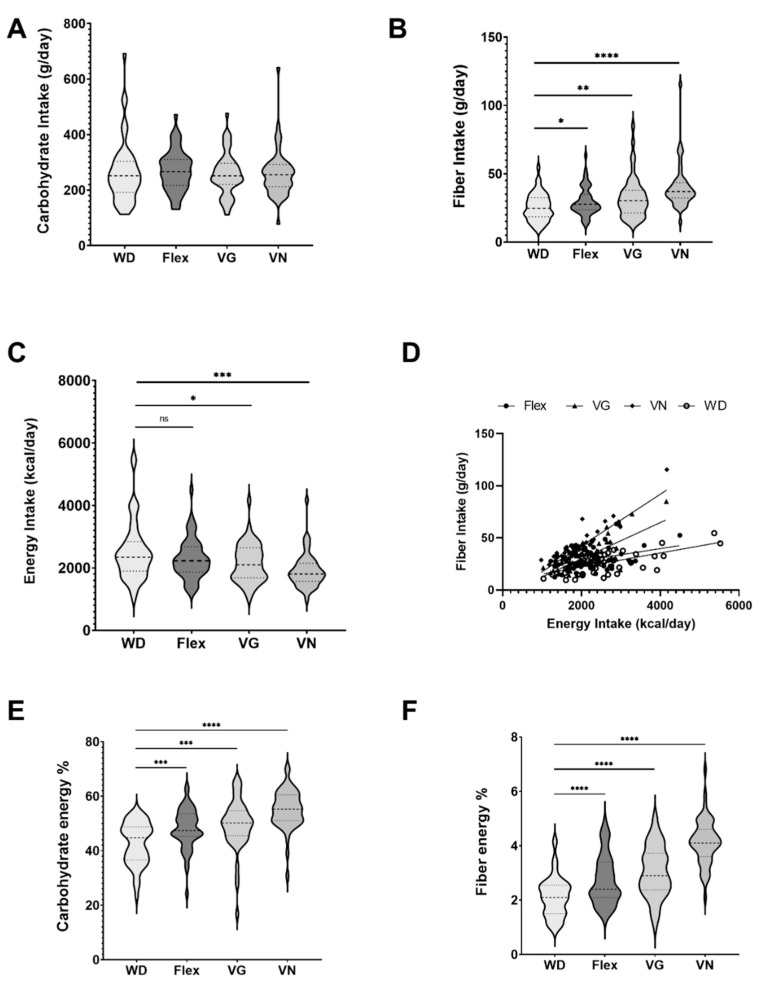
Differences in carbohydrate and fiber intake (g/day) as well as energy intake (kcal/day) for each dietary group (**A**–**C**). Regression of energy intake and fiber intake (**D**). Percentage of daily energy consumption of carbohydrates (**E**) and fiber (**F**); ns: not significant, *: *p* < 0.05, **: *p* < 0.01, ***: *p* < 0.001, and ****: *p* < 0.0001.

After assessing the interaction of alpha and beta diversity with the corresponding diet types, we looked at possible effects of carbohydrate, fiber, and energy intake on the baseline microbiome. For absolute carbohydrate intake (g/day), we did not observe significant differences across all diets (Table 5) or for pairwise comparisons against WD—the reference diet (Figure 3A). In contrast, the pairwise comparison of the percentage of daily energy intake of either carbohydrates or fiber were highly significant compared to the WD (Figure 3E,F). WD and VN were found to be opposite extremes regarding fiber intake (Figure 3B) and energy intake (Figure 3C). Vegans showed the highest fiber intake and the lowest energy intake, whereas the WD displayed the opposite. The pairwise difference between WD and VN, for these two points, was highly significant (energy intake, *p* < 0.001; fiber intake, *p* < 0.0001; Figure 3). For all groups, a highly significant correlation between energy intake and fiber intake (strong correlation r > 0.63) for WD, VN, and VG and a moderate correlation (r = 0.37) for Flex were observed; linear regression shown in Figure 3D).

Table 5 presents the data based on the one-way ANOVA test across all diets. There was a highly significant difference between energy intake and fiber intake between the investigated diet groups, with dietary fiber showing a high effect size and energy intake a medium effect size. Following the results, the plant-based diets are associated with a reduction of body weight and body fat percentage [2].

The results reported here show no correlation between alpha diversity metrics and carbohydrate intake in g/day in the studied diet groups. Correlation tests were also performed for each dietary pattern. Significant correlations were only found for WD and VN.

A highly significant, moderate, and inverse correlation between carbohydrate intake (g/day) and alpha diversity metrics was detected for the VN group. These data correspond to a decrease in bacterial diversity with increasing carbohydrate consumption in the VN diet, as shown in Table 6. The opposite was observed for WD, where a medium correlation of the Faith-PD and carbohydrate intake suggests increased phylogenetic diversity of the gut microbiome with increasing carbohydrate consumption (Table 6). No correlation was found for both flexitarians and vegetarians.

Similar relations were detected for fiber intake and alpha diversity. No significant correlation was found between fiber intake in all dietary groups and alpha diversity metrics (Table 7). The fiber intake in the vegan group correlates significantly with a moderate reduction of all alpha diversity metrics (Shannon entropy, Pielou evenness: *p* < 0.05; Table 7). No correlations were observed in the flexitarian and the vegetarian group (Table 7). In contrast, an increased fiber consumption in WD expresses itself in a more evenly distributed microbiome (Pielou evenness: *p* < 0.05; Table 7).

An inverse correlation between energy intake and alpha diversity was detected in the vegan group (*p* < 0.05; Table 8). This data suggests for reduction of microbiome diversity with increased energy intake in the vegan group. No further correlations were found between energy intake and alpha diversity (Table 8).

Comparable correlations between intake of single fiber subgroups such as non-soluble fiber, cellulose, and starch and alpha diversity were also found in the WD group and vegans (Table 9). Interestingly, the negative correlation between intake of fiber compounds and alpha diversity was not observed for soluble fiber (Table 9).

The differences observed in mono-, di-, and oligosaccharides were smaller (Table 10). For monosaccharides, we detected a positive correlation with alpha diversity in WD, Flex, and VG (*p* < 0.05; Table 10). For disaccharides, we observed a negative correlation only in the vegan group (*p* < 0.05; Table 10). In vegans, the negative correlation between intake of oligosaccharides and alpha diversity was not significant (Table 10).

### 3.4. Dietary Impact on Taxonomy of the Gut Microbiome

Based on the total relative abundances across all diets, the ten most abundant genera averaged across each diets were: Bacteroides (in order WD/Flex/VG/VN: 12.8/16.1/18.0/18.9%), Blautia (8.9/8.4/7.3/7.9%), Fecalibacterium (7.0/7.1/6.9/7.8%), Prevotella (7.2/6.5/4.7/4.1%), Agathobacter (3.0/3.3/2.4/3.8%), Bifidobacterium (2.1/2.9/3.4/3.1%), Anaerostipes (2.8/3.0/2.5/2.9%), Subdoligranulum (2.8/2.7/2.7/2.7%), Alistipes (2.2/2.3/3.0/2.5%), and Roseburia (2.0/2.0/2.5/3.3%). These genera comprised approximately 50% of the fecal microbiome, whereas the remaining 91 genera (after filtering) made up the remaining 50% (Supplementary Figure S2; Supplementary Table S1).

**Figure 4:**
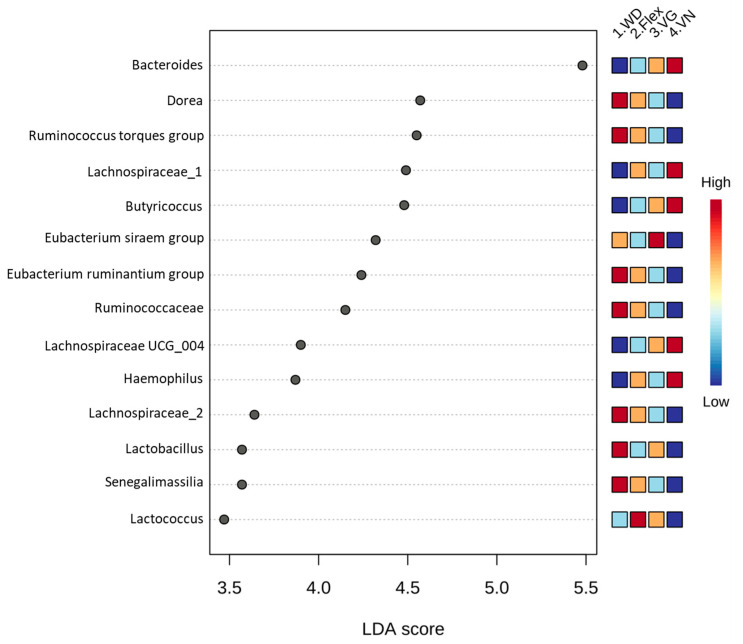
LefSe analysis was done in MicrobiomeAnalyst with the filtered data set. The four diets were set as color-coded abundancy classes (high = red to low = blue). Fourteen significant biomarkers (*p* < 0.05) were identified. LDA score = linear discriminant analysis score.

**Figure 5:**
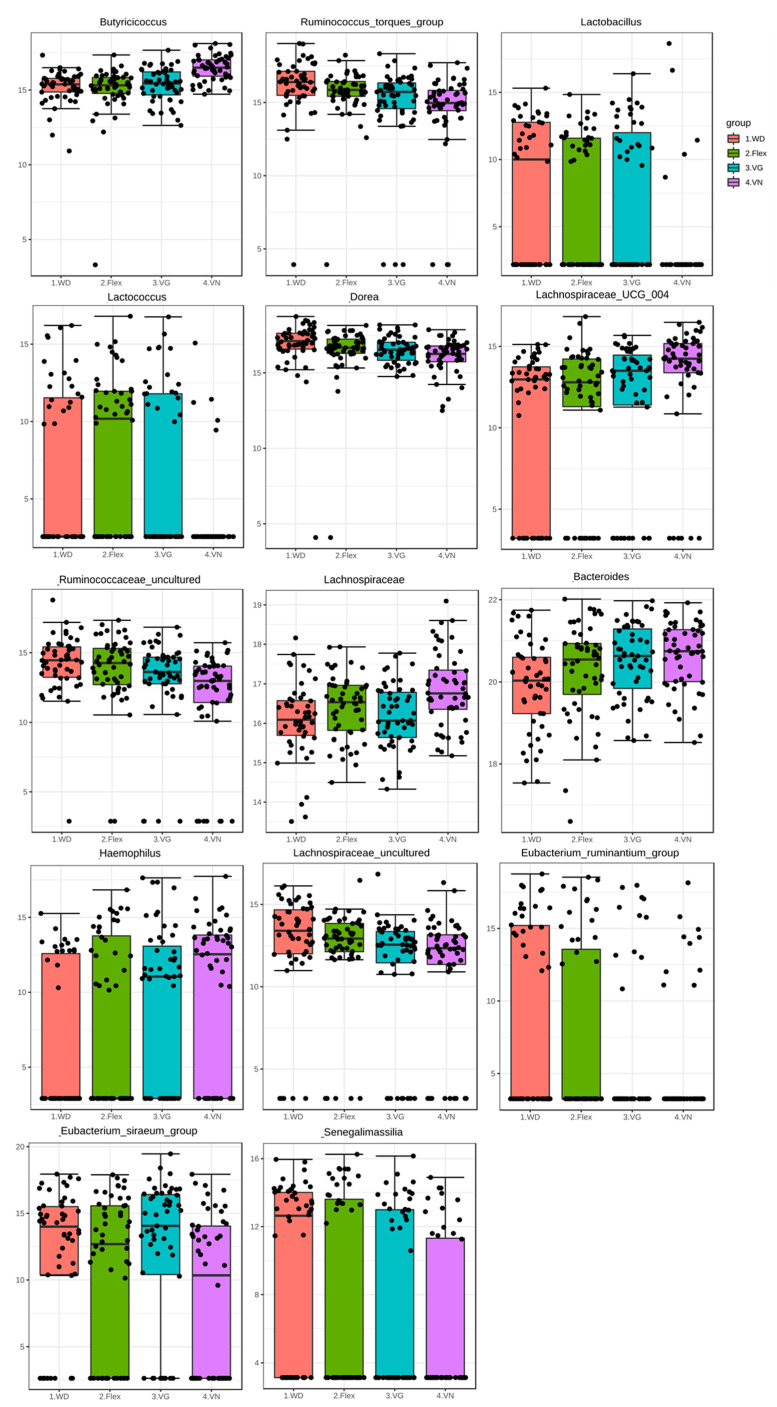
Log-transformed count of the 14 significant biomarkers based on the LefSe analysis. Bar plots with individual sample points are depicted for each diet.

A LefSe analysis was performed to determine significant biomarkers on a genus level (if possible) for the respective diet and showed the most prominent differences between WD and vegan dietary patterns. For Bacteroides, Lachnospiraceae_1, Butyricoccus, Lachnospiraceae UCG_004, and Haemophilus, the lowest abundance was observed in the WD group, and the highest abundance was seen in vegans (Figure 4 and Figure 5). For Dorea, Ruminococcus torques group, Eubacterium ruminantium group, Ruminococcaceae, Lachnospiraceae_2, Lactobacillus, and Senegalimassilia, the highest abundance was found in the WD group, and the lowest abundance was analyzed in vegans (Figure 4 and Figure 5). A striking observation was observed in the Eubacterium siraem group: the abundance differed in both plant-based groups with the lowest abundance in VN and the highest in VG. For Lactococcus, the lowest abundance was found in vegans and the highest was observed in flexitarians. As expected, Lactobacillus and Lactococcus were not found in the most vegan participants nor was the Eubacterium ruminantium group detected in most of the vegetarians and vegans (Figure 4 and Figure 5).

**Figure 6:**
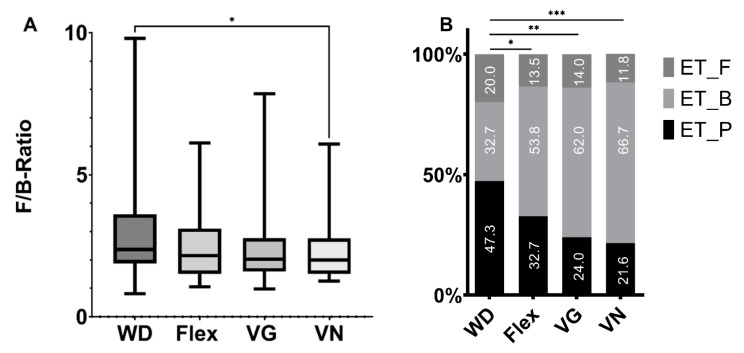
(**A**) Firmicutes/Bacteriodetes ratio distribution for each diet. ANOVA showed no significant difference across all diets. Pairwise comparison was only significant for WD versus VN (*p* < 0.05). (**B**) Enterotypes according to Le Chatelier et al. [20]. ET_F = Firmicutes-enriched; ET_B = Bacteroides-enriched; ET_P = Prevotella-enriched. Across all groups, the composition of the enterotypes was statistically different, as calculated by ANOVA with a *p*-value of 0.005. * shows corresponding significance levels (* *p* < 0.05, ** *p* < 0.01, *** *p* < 0.001) of the pairwise comparisons obtained by *t*-tests.

For a further high-level analysis, we also calculated the Firmicutes/Bacteriodetes ratio distribution for each diet (Figure 6A). Over all diets, no significant difference could be observed. A pairwise comparison found a significant difference only for WD versus VN (*p* < 0.05). Enterotype analysis was yet another option. Here we could see a gradual shift in the distribution of the three enterotypes (Firmicutes, *Bacteroides*, *Prevotella*). The WD showed the most homogenous distribution, with *Prevotella*-enriched samples accounting for the largest proportion, followed by *Bacteroides*-enriched and Firmicutes-enriched samples. Then progressing in a gradient from WD to Flex to VG to VN, the proportion of *Bacteroides*-enriched samples increases from 33% up to 67%, whereas the other two enterotypes decreased down in vegans to 12% (Firmicutes-enriched) and 22% *(Prevotella*-enriched), respectively (Figure 6B). Across all groups, the composition of the enterotypes was statistically different, as calculated by ANOVA with a *p*-value of 0.005. In the pairwise comparison with WD, the enterotype composition of each diet was also significantly different (WD—Flex, *p* = 0.029; WD—VG, *p* = 0.009; WD—VN, *p* = 0.001; Figure 6B).

These data show, especially for the WD and Flex diets, that these dietary patterns can result in different gut microbiome compositions, indicating the need for a more personalized approach for diet recommendations in connection with the microbiome.

**Figure 7:**
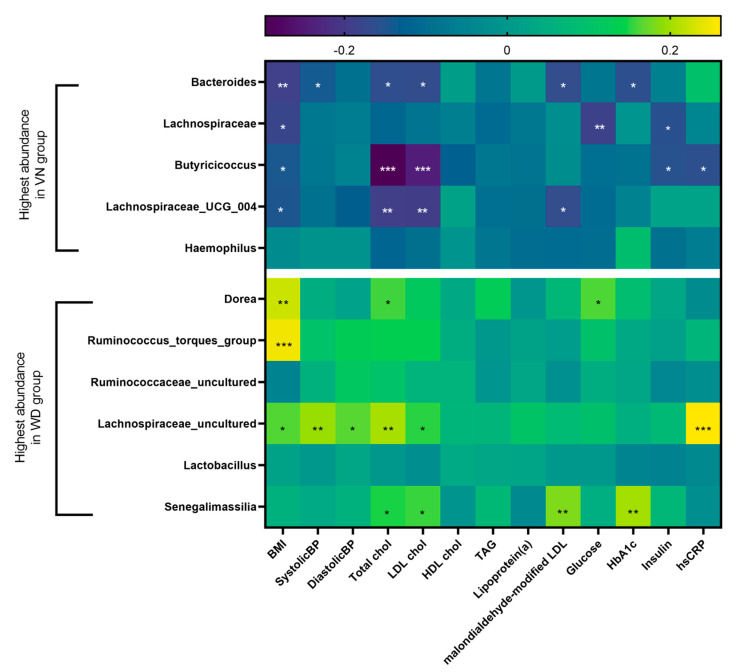
Heatmap of the correlation analysis of 11 significant genera, separated by maximum abundance in WD or VN group, resulting from the LefSe analysis of the four diets tested. Top legend represents corresponding r-values. Significant results are marked as following: *: *p* < 0.05, **: *p* < 0.01, ***: *p* < 0.001. BMI = body mass index; BP = blood pressure; Total chol = total cholesterol; LDL chol = low-density lipoprotein cholesterol, HDL chol = high-density lipoprotein cholesterol; TAG = triacylglycerides; HbA1c = hemoglobin A1c; hsCRP = high sensitivity *c*-reactive protein.

Based on the LefSe analysis results, we separated the genera according to the maximum abundances in WD and VN groups, respectively, and performed a correlation analysis using anthropometric data, blood pressure, and other blood parameters (cardiovascular risk factors). Eleven genera significantly correlated with body mass index (BMI), blood pressure, total cholesterol, low-density lipoprotein (LDL) cholesterol, malondialdehyde-modified LDL, blood glucose, hemoglobin A1c (HbA1c), insulin, and high-sensitivity reactive protein (hsCRP; Figure 7). No significant correlations were observed for high- density lipoprotein (HDL) cholesterol, triacylgycerides (TAG), and lipoprotein(a). The color coding already shows that the VN-specific genera tend to show a negative correlation with the above-mentioned values, whereas the WD-specific genera tend to show a positive correlation. For example, BMI is negatively correlated with four out of five VN-specific genera, whereas three out of six WD-specific genera correlated positively with BMI. VN-specific genera Butyricicoccus (*p* < 0.001), Lachnospiraceae UCG_004 (*p* < 0.01), and Bacteroides (*p* < 0.05) showed a significant negative correlation with total and LDL cholesterol. In addition, Bacteroides also showed negative significant correlations with malondialdehyde-modified LDL and HbA1c (*p* < 0.05). An unknown species of Lachnospiraceae shows a negative correlation with glucose (*p* < 0.01) and insulin (*p* < 0.05), and Butyricicoccus correlates negatively with insulin and hsCRP (*p* < 0.05).

No significant correlations were observed for Haemophilus.

As a WD representing genus, an uncultured genus of the family of Lachnospiraceae showed the most amount of significant positive correlations, including values for BMI, blood pressure, total cholesterol, LDL cholesterol, and hsCRP (*p* < 0.05). Senegalimassila showed a positive correlation with total and LDL cholesterol, malondialdehyde-modified LDL, and HbA1c. Moreover, Dorea and Ruminococcus torques group showed highly significant positive correlations with BMI (*p* < 0.01; Figure 7).

## 4. Discussion

Personalized nutrition assumes that each person may have a different response to specific foods and nutrients and will benefit from individual concepts adapted to their health status and lifestyle factors. This concept also considers the microbiome and its influence on our metabolism. The human microbiota has numerous functions playing a pivotal role in health maintenance. It comprises all microbial taxa associated with human beings such as bacteria, viruses, fungi, protozoa, and archaea. It is assumed that 38 × 1012 (trillion) bacterial cells are harbored by each person [21]. It is hypothesized that its composition is unique to everyone. In daily practice, the development and implementation of individual and personalized nutrition concepts is a great challenge.

One approach to addressing this problem may be the development of concepts for individuals with the common metabolism-influencing characteristics such as age, gender, physical activity, and diet. We have shown that diet has a strong impact on types of bacteria colonizing our digestive tracts [22]. For example, consumption of the Mediterranean diet for one year by obese men (*n* = 20) significantly decreased the genera Prevotella and increased Roseburia and Oscillospira [23]. Roseburia is a known butyrate producer with immune maintenance and anti-inflammatory properties [24]. Furthermore, the implementation of different long-term dietary patterns, such as plant-based diets vs. omnivorous diets, significantly influences our gut microbiota composition [25]. The findings of the NuEva study support the idea that diet strongly influences microbiome composition. This is most evident in omnivores and vegans (Figure 4 and Figure 5).

The vegan diet has a lower energy value but is comparably rich in dietary fiber. The gut microbiota plays an important role in the fermentation of these non-digestible substrates to SCFAs, which are important signaling molecules [9,10]. Hence, it could be conceivably hypothesized that different intakes of dietary fibers have varying impacts on human gut microbiota. Following this hypothesis, Firmicutes (in order WD/Flex/VN/VG) such as Fecalibacterium (7.0/7.1/6.9/7.8%), Roseburia (2.0/2.0/2.5/3.3%), and Anaerostipes (2.8/3.0/2.5/2.9%), which are famous for producing butyrate, belong to the 10 most abundant genera for all diet forms (Supplementary Figure S2). The abundance of two from these three is higher in vegans, indicating a higher production of butyrate. Moreover, the lowest abundance of Lachnospiraceae_1 and Lachnospiraceae UCG_004 was observed in the WD group and the highest abundance was seen in vegans (Figure 4 and Figure 5). The high abundance of Lachnospiraceae, a family of obligate anaerobic and variably spore-forming bacteria in the order Eubacteriales, in vegans points to high fermentation of plant-based polysaccharides to SCFAs such as butyrate, acetate, and alcohols [26]. Butyrate is an important energy source for the cells of the gut lining. It has further health benefits such as its antioxidant properties that stimulate the production of glutathione, controlling intestinal inflammation and protecting against colon cancer in humans [27,28]. The higher abundance of Lachnospiraceae in vegans compared to omnivores was also reported by other groups [29,30].

However, the high abundance of Bacteroides detected in the vegan group contrasts with data from Wu et al. [31] that described two diet-related enterotypes. The first enterotype dominated by Bacteroides is adapted to diets high in protein and animal fats, while the Prevotella enterotype is associated with carbohydrate metabolism and a vegetarian diet. Bacteroides are related to a more pro-inflammatory state and possibly related to the increased risk of the metabolic syndrome and further pathological conditions [25]. In contrast, the systematic review of Losno et al. [32] observed an increase in Bacteroidetes on the phylum level and a higher abundance of Prevotella on the genus level in vegans compared to omnivores. The higher abundance of Bacteroidetes in our data reflected Losno‘s findings [32]. However, an increase of Prevotella in VG or VN was not evident in the NuEva collective (Supplementary Figure S2). Furthermore, the review of Zafar and Saier [33] showed that specific Bacteroides species primarily depend on adults’ dietary pattern. In general, Bacteroides species (spp.) are involved in the digestion of various polysaccharides [34], provide protection from pathogens, and supply the host and other microbial cells with nutrients. Higher percentages of B. thetaiotaomicron, B. salyersiae, as well as B. vulgatus are found in vegetarians and vegans, whereas B. fragilis, B. salanitronis, and B. coprocola are more prevalent in omnivores. In our study, the most identified Bacteroides species were B. vulgatus (WD, 4.1%; Flex, 4.3%; VG, 6.0%; VN, 6.7%, on average), B. uniformis (WD, 2.6%; Flex, 3.2%; VG, 3.2%; VN, 3.0%), B. thetaiotaomicron (WD, 0.3%; Flex, 0.4%; VG, 0.5%; VN, 0.4%) and B. cellulosilyticus (WD, 0.2%; Flex, 0.6%; VG, 0.4%; VN, 0.4%).

In contrast, several studies to date reported higher abundance of Prevotella in vegans compared to omnivores [29,35,36].

Previous research results on Bacteroides have been inconsistent and contradictory. Some recent studies have observed higher levels of Bacteroides in vegans [35,37], while others have reported a lower abundance of Bacteroides in vegans compared to omnivores [35,38].

*Prevotella* spp. Are associated with anti-inflammatory disorders, such as rheumatoid arthritis and multiple sclerosis. *Bacteroides* spp. Have been involved in several infections by exhibiting antimicrobial resistance to a variety of antibiotics. Furthermore, they may act as useful commensals to the human host [39].

The lowest abundance of Haemophilus was detected in the WD group and the highest abundance was seen in vegans (Figure 4 and Figure 5). If detected, the counts found for Haemophilus were in the same size range for all diets, and the median was largely driven by the presence or absence of Haemophilus (Figure 5). Recent publications suggest negative health effects. Zhu et al. [40] found a significant correlation between Haemophilus and psychiatric disorders such as schizophrenia, depression, and impacts on patients’ cognition and depression. The microbiome of the respiratory tract is also known to affect immune response. Here, increased levels of Haemophilus are linked to mucosal inflammation [41]. Amongst genera such as Staphylococcus, Moraxella, and Pseudomonas, Haemophilus seems to promote pro-inflammatory Th2 and Th17 cytokines and correlate with higher levels of IgE [42]. Further investigation needs to be done to identify possible dietary factors that may influence the prevalence of Haemophilus as part of the gut microbiome and if this genus could be a negative biomarker in relation with high carbohydrate/fiber consumption.

Conversely, we observed the highest abundance of Dorea, Ruminococcus torques group, Eubacterium ruminantium group, Ruminococcaceae, Lachnospiraceae_2, Lactobacillus, and Senegalimassilia in the WD group, while these species are lowest in vegans (Figure 4 and Figure 5).

In contrast to our data, Singh et al. [43] reported that the intake of animal protein alters gut microbiota composition by increasing *Bacteroides* spp., *Alistipes* spp., and *Bilophila* spp., and decreasing beneficial *Lactobacillus* spp., *Roseburia* spp., and Eubacterium rectale. De Angelis et al. [30] and De Fillippis et al. [29] also reported that Ruminococcus and the family of Ruminococcaceae were positively associated with an omnivorous diet. Additionally, higher amounts of lactic acid bacteria, including Lactobacillus and Lactococcus, were found in omnivores compared to vegans [7]. A regular consumption of whole grain and wheat bran was associated with an increase of *Bifidobacterium* spp. And *Lactobacillus* spp., whereas resistant starch and whole grain barley seemed to increase lactic acid bacteria including *Ruminococcus* spp., Eubacterium rectale, and *Roseburia* spp. [39]. Furthermore, a long-term consumption of fruit and vegetables as well as walnuts affects the gut microbiota composition by increasing *Ruminococcus* spp. And *Bifidobacterium* spp. And decreasing *Clostridium* spp. [30,44]. These findings, however, are not supported by the current study as these foods are consumed in both diets, with much higher intakes in vegans [2]. Ruminococcus might also play a role in the conversion of animal-derived choline to trimethylamine (TMA) [45]. Thus, the abundance of Ruminococcus is influenced by both animal and plant-based diets.

Fat intake was the lowest in vegans, favoring beneficial Bifidobacteria in human gut microbiota, which is in accordance with our data (Bifidobacterium in WD/Flex/VG/VN: 2.1/2.9/3.4/3.1%). Previous studies found a lower abundance of both Bifidobacteria and Enterobacteria in vegans [35,37,38].

In a normal gut flora, Bacteroidetes and Firmicutes represent approximately 90% of total bacterial phyla constitution, while Actinobacteria, Proteobacteria, and Verrucomicrobia are represented to a smaller extent [39]. In plant-based diets, the fat content consists predominantly of mono- and polyunsaturated fatty acids which is related to a decrease in Firmicutes/Bacteriodetes ratio, and on the genera level, increase in lactic acid bacteria, Bifidobacteria, and Akkermansia muciniphila [43]. In the NuEva collective, the higher Firmicutes/Bacteriodetes ratio in the WD group differed significantly from the lower ratio in vegans. A decrease in Firmicutes/Bacteriodetes ratio was also seen in response to an increased intake of resistant starches, which may support a weight management hypothesis [46]. Recent data are inconsistent as De Fillippis et al. [29] observed a higher ratio of Firmicutes to Bacteroidetes in omnivores, while no significant difference in this ratio was detected by Losasso et al. [47].

Enterotypes present an initial form of pattern analysis of the gut microbiome and were introduced by Arumugam et al. [48]. The intestinal microbiome is divided into three groups: ET_B = Bacteroides, ET_P = Prevotella, and ET_F = Firmicutes enterotypes. It was traditionally reported that Prevotella was strongly associated with a carbohydrate-rich diet. Protein and animal fat consumption would result in a Bacteroides enterotype. In our study, high carbohydrate/fiber consumption typical of vegetarian and vegan dietary patterns resulted in an increase of identified Bacteroides enterotypes in comparison to WD and Flex (Figure 6B). This contradicts the traditional classification but is not unique. Jang et al. [49] described similar results for their adult Korean cohort. They found diets based on plants and fermented foods are associated with higher proportions of Bacteroides and lower proportions of Prevotella relative to a WD. The animal-based foods of the WD had a stronger association with Prevotella and were related to a higher risk of obesity. A possible explanation for these contradictory observations could be found in the publication of Méndez-Salazar et al. [50]. They investigated compositional changes of the gut microbiome in obese and undernourished children. The obese as well as the undernourished group showed lower bacterial richness and diversity than the normal-weight group. These results are in accordance with this study‘s observed alpha diversity metrics, which were reduced for vegans in comparison to WD/Flex groups. In the NuEva collective, the energy intake and the BMI were significantly low in vegans (*p* < 0.05), [2]. Interestingly, they described different associations of nutrient intake and the Bacteroides enterotype. For the obese group, the Bacteroides enterotype correlated positively with dietary fat intake, whereas for the undernourished group the Bacteroides enterotype correlated positively with carbohydrate intake. These data indicate that dietary habits differ in terms of their impact on the microbiome depending on individual baseline conditions.

Some previous studies detected greater microbial diversity in certain Bacteroidetes-related operational taxonomic units in plant-based diets or diets characterized by long-term fruit and vegetable intake [47,51]. In addition, the intake of whole-grain barley and brown rice increased microbial diversity in healthy volunteers [52]. Losasso et al. [47] found also a significantly lower alpha diversity in omnivores compared to vegans. Other studies did not report a significant difference in alpha diversity between vegans and omnivores [29,31]. According to our results, Trefflich et al. [36] observed a lower alpha diversity in vegans compared to omnivores. The increased carbohydrate consumption observed in vegans may be attributed to a limited food diversity. WD is characterized by a high intake of animal-based foods. An increase in the proportion of carbohydrates is usually accompanied by an increase in the proportion of plant-based foods. This increase in food diversity could have a favorable effect on the parameters studied and could explain the opposing trend in alpha diversity among both diet groups.

The data available from the NuEva collective show great differences in the energy and nutrient intake between vegans and omnivores, whereby the intakes in both diets are inversely related [2]. This, in turn, has an impact on microbiome composition (Figure 4 and Figure 5).

The data on alpha and beta diversity account for the difference in the fecal bacterial composition between high-fiber and low-fiber dietary patterns. This was also observed within diet groups. Together with the observed reduction in alpha diversity in the vegan group, we conclude that limited food choices and the extremely high intake of dietary fibers result in a reduction in the microbial diversity (Table 7 and Table 9). Interestingly, the intake of soluble fibers in vegans was not related to a reduction in the microbial diversity (Table 9). The higher intake of soluble fibers in vegans (Median/IQR: 10.1/4.6 g/day; 1.8–30.8 g/day) differs significantly from the other three diet groups (7.9–9.0 g/day; 3.3–24.7 g/day, *p* < 0.05) [2]. A regular intake of soluble fiber-rich foods such as oats, whole barley, legumes, peas, beans, flax seeds, apples, and citrus foods was associated with a reduction in total and low-density lipoprotein cholesterol levels by about 5–10% [53,54]. This is in line with our data [2].

No correlations between dietary fiber intake and alpha diversity were observed in flexitarians and vegetarians. An increased fiber consumption in WD expresses itself in a more evenly distributed microbiome (Table 7 and Table 9). This is in accordance with latest findings that described a relation between dietary fiber intake and overall metabolic health, in particular through key pathways that include insulin sensitivity. In addition, a reduction in cardiovascular risk factors, such as blood lipids (mainly due to consumption of soluble fibers), body weight, and abdominal adiposity was reported. Dietary fiber intake is also related to a reduction in mortality and colorectal carcinoma risk. In this context, the gut microflora mediates health benefits of dietary fiber, including the regulation of appetite, metabolic processes, and chronic inflammatory pathways [55].

On the other hand, a high intake of dietary fibers is associated with a high intake of phytic acid, which has a high affinity to chelated Zn^2+^ and Fe^2+^, Mg^2+^, Ca^2+^, K^+^, and Mn^2+^ and Cu^2+^. Consequently, the trace elements and minerals chelated in phytic acid are not bioavailable. Given the fact that these micronutrients are critical nutrients, especially in plant-based diets, the reduction in their bioavailability increases the risk for micronutrient deficiencies affecting growth, development, and overall health [56].

Regardless of the fiber group, vegans showed significantly different microbiome compositions than the WD group (Table 4), indicating that the microbiome may not be affected by single macronutrients but rather must be assessed in the context of overall nutrition. In the NuEva collective, reduced intake of energy, fat, and saturated fatty acids and an increased intake of dietary fibers in vegans resulted in a reduction in total cholesterol and LDL cholesterol, both of which are established risk factors for cardiovascular diseases [2,57]. A LefSe analysis allowed us to identify 14 diet-specific biomarkers at the genus level. Of these, 11 showed minimum or maximum counts in the respective “extreme” diet groups WD and VN. In vegans, we found mainly fermenters (Lachnospiraceae, Butyricoccus, Lachnospiraceae UCG_004), which correlated negatively with cardiovascular risk factors such as BMI, blood pressure, and blood lipid values (Figure 7). In contrast to previous studies, we detected Bacteroides as the main enterotype in vegans, showing similar behavior to the other VN-specific biomarkers. This discrepancy also illustrates here that it is not always possible to make a generally valid statement.

On the other hand, WD-specific biomarkers showed mostly positive correlations with BMI and blood lipids. These include Dorea, a genus of harmful bacteria. Current studies also show a link to insulin resistance, and this genus is also considered as a risk marker for colorectal cancer and irritable bowel syndrome [58]. Unexpectedly, an uncultured genus of Lachnospiraceae was shown to be a biomarker for WD, which also revealed several significant positive correlations with BMI, blood pressure, and total and LDL cholesterol. Bacteria from this genus are known to produce SCFAs, which are associated with a positive impact on gut health. Lactobacillus was also identified as a biomarker for WD but did not show significant correlations. Its identification in some vegans could result from consumption of fermented vegetables such as sauerkraut or kimchi, as well as miso or kombucha.

Apart from the described differences in the intake of energy and dietary fibers in the NuEva collective, the amount and composition of protein and fat differs also markedly between the WD groups and vegans [2]. Further studies which take the effects on the diversity and composition of the microbiome into account will need to be undertaken.

## 5. Conclusions

This study has shown the distinction between energy intake and consumption of dietary fibers between omnivores (WD) and vegans. This disparity is reflected in differences in the microbiome profile. In addition, we found a negative correlation between alpha diversity and energy, carbohydrates, and dietary fiber intake only in the vegan group. Our data indicate that the comparable high intake of dietary fibers in vegans leads to a restriction of the diversity of the microbiome. Interestingly, this relation does not apply to the intake of soluble fiber. In addition, the high intake of dietary fibers may reduce the bioavailability of trace elements and minerals but also reduce cardiovascular risk factors.

The findings reported here shed new light on the advantages and disadvantages of the studied diets. Identifying biomarkers for the diets on the extreme end of the spectrum (WD and VN) and their association with cardiovascular risk factors highlights the potential and the need for the development of personalized recommendations depending on dietary patterns. Our data both confirm and contradict the findings of other studies, underscoring the need for further research. In addition, several questions on the meaning of the detected diet-specific differences in microbiome composition cannot be adequately answered. The elucidation of these associations will form the basis for personalized nutritional recommendations based on the microbiome.

Regarding fiber intake, our data indicate that optimal diversity of the human microbiome can be achieved by an increase in the WD and an upward restriction for vegans.

Conversely, the high intake of dietary fiber in vegans, soluble fiber in particular, is associated with a reduction in cardiovascular risk factors such as BMI, blood pressure, and blood lipids. To maintain these favorable effects, strategies to increase the variety of food sources within a particular dietary lifestyle are urgently needed.

For vegans, the recommendation to include all varieties of vegetables, pulses, whole-grains, nuts, seeds, and micro- and macroalgae, as well as plant-based oils and fermented foods, seems to be a promising opportunity. To increase bioavailability of the nutrients, the usage of appropriate methods of preparation is also advisable.

For WD, partial replacement of animal foods with plant foods is similarly advisable.

Both strategies based on the habitual diet form will contribute to reducing cardiovascular risk factors and increase the diversity of the microbiome.

## Figures and Tables

**Table 1:** Difference of alpha diversity across all diet types (WD, Flex, VG, VN) and between diet types (Flex, VG, VN) compared to the reference diet (WD).

	ANOVA Across All Diets		Two Sample *t*-Test					
	(*n* = 206)		Flex vs. WD (*n* = 52)		VG vs. WD (*n* = 51)		VN vs. WD (*n* = 52)	
Alpha Diversity	Effect Size	*p*-Value	Effect Size	*p*-Value	Effect Size	*p*-Value	Effect Size	*p*-Value
**Shannon Entropy**	medium	0.082	small	0.188	small	0.114	medium	0.011
**Pielou’s Evenness**	small	0.266	small	0.118	medium	0.057	small	0.137
**Faith PD**	small	0.166	small	0.435	small	0.398	medium	0.031

WD = Western Diet, Flex = Flexitarian, VG = Vegetarian, VN = Vegan, Faith PD = Faith’s phylogenetic diversity; Significance levels: *p* < 0.05 significant, *p* < 0.01 highly significant.

**Table 2:** PERMANOVA statistical analysis of beta diversity metrics across all groups as well as in pairwise comparison against WD/reference diet.

	PERMANOVA Across All Diets	Pairwise PERMANOVA Results		
	(*n* = 205)	Flex vs. WD (*n* = 102)	VG vs. WD (*n* = 101)	VN vs. WD (*n* = 102)
Beta Diversity	*p*-Value	*p*-Value	*p*-Value	*p*-Value
**Unweighted Unifrac**	0.015	0.142	0.034	0.002
**Weighted Unifrac**	0.011	0.247	0.013	0.003
**Bray-Curtis**	0.001	0.211	0.001	0.001
**Jaccard**	0.001	0.163	0.006	0.001

WD = Western Diet; Flex = Flexitarian; VG = Vegetarian; VN = Vegan. Significance levels: *p* < 0.05, significant; *p* < 0.01, highly significant.

**Table 3:** Results of the pairwise PERMANOVA statistical analysis of beta diversity metrics between high and low fiber content groups of the same diet.

	Pairwise PERMANOVA Results			
	WD-High vs. WD-Low (*n* = 50)	Flex-High vs. Flex Low (*n* = 52)	VG-High vs. VG-Low (*n* = 51)	VN-High vs. VN-Low (*n* = 52)
Beta Diversity	*p*-Value			
**Unweighted Unifrac**	0.355	0.360	0.130	0.083
**Weighted Unifrac**	0.113	0.132	0.330	0.151
**Bray-Curtis**	0.100	0.205	0.034	0.268
**Jaccard**	0.112	0.280	0.039	0.154

WD = Western Diet; Flex = Flexitarian; VG = Vegetarian; VN = Vegan. Significance levels: *p* < 0.05, significant; *p* < 0.01, highly significant.

**Table 4:** Results of the pairwise PERMANOVA statistical analysis of beta diversity metrics comparison against WD/reference diet split between high fiber and low fiber content groups.

		Pairwise PERMANOVA Results		
		Flex vs. WD (*n* = 33/69)	VG vs. WD (*n* = 42/59)	VN vs. WD (*n* = 57/45)
	Beta Diversity	*p*-Value		
**Fiber high**	**Unweighted Unifrac**	0.286	0.441	0.030
	**Weighted Unifrac**	0.407	0.323	0.026
	**Bray-Curtis**	0.748	0.126	0.004
	**Jaccard**	0.543	0.149	0.006
**Fiber low**	**Unweighted Unifrac**	0.173	0.054	0.071
	**Weighted Unifrac**	0.112	0.008	0.049
	**Bray-Curtis**	0.209	0.003	0.001
	**Jaccard**	0.337	0.080	0.043

WD = Western Diet; Flex = Flexitarian; VG = Vegetarian; VN = Vegan. Significance levels: *p* < 0.05, significant; *p* < 0.01, highly significant.

**Table 5:** One-way ANOVA test of energy intake, carbohydrate intake, and fiber intake across all dietary forms.

	*F*	Effect Size *f* (num)	Effect Size (categ)	*p*-Value
**Energy intake (kcal/day**)	6.8	0.32	medium	2.2 × 10^−4^
**Carbohydrate intake (g/day)**	0.2	0.06	small	0.88
**Carbohydrate energy %**	21.8	0.57	high	2.9 × 10^−12^
**Fiber energy %**	31.5	0.83	high	5.6 × 10^−16^
**Fiber intake (g/day)**	11.7	0.42	high	4.5 × 10^−7^

**Table 6:** Pearson correlation between carbohydrate intake (g/day) and alpha diversity in diet types.

	Overall (*n* = 205)		WD (*n* = 50)		Flex (*n* = 52)		VG (*n* = 51)		VN (*n* = 52)	
**Alpha Diversity**	*r*	*p*-Value	*r*	*p*-Value	*r*	*p*-Value	*r*	*p*-Value	*r*	*p*-Value
**Shannon Entropy**	0.02	0.801	0.22	0.124	0.07	0.616	0.14	0.320	−0.38	0.005
**Pielou Evenness**	−0.01	0.939	0.14	0.323	0.14	0.341	0.08	0.601	−0.38	0.005
**Faith PD**	0.003	0.670	0.34	0.017	0.01	0.947	0.13	0.357	−0.38	0.005

WD = Western Diet; Flex = Flexitarian; VG = Vegetarian; VN = Vegan; Faith PD = Faiths’s phylogenetic diversity. Significance levels: *p* < 0.05, significant; *p* < 0.01, highly significant.

**Table 7:** Pearson correlation between fiber intake (g/day) and alpha diversity in diet types.

	Overall (*n* = 205)		WD (*n* = 50)		Flex (*n* = 52)		VG (*n* = 51)		VN (*n* = 52)	
**Alpha Diversity**	*r*	*p*-Value	*r*	*p*-Value	*r*	*p*-Value	*r*	*p*-Value	*r*	*p*-Value
**Shannon Entropy**	−0.09	0.198	0.20	0.173	0.07	0.619	0.08	0.565	−0.31	0.023
**Pielou Evenness**	−0.04	0.573	0.30	0.036	0.17	0.235	0.04	0.762	−0.28	0.046
**Faith PD**	−0.12	0.082	0.11	0.447	−0.08	0.585	0.05	0.726	−0.26	0.065

WD = Western Diet; Flex = Flexitarian; VG = Vegetarian; VN = Vegan; Faith PD = Faiths’s phylogenetic diversity. Significance levels: *p* < 0.05, significant; *p* < 0.01, highly significant.

**Table 8:** Pearson correlation between energy intake (kcal/day) and alpha diversity in diet types.

	Overall (*n* = 205)		WD (*n* = 50)		Flex (*n* = 52)		VG (*n* = 51)		VN (*n* = 52)	
**Alpha Diversity**	*r*	*p*-Value	*r*	*p*-Value	*r*	*p*-Value	*r*	*p*-Value	*r*	*p*-Value
**Shannon Entropy**	0.01	0.872	0.07	0.620	−0.02	0.877	0.11	0.434	−0.42	0.002
**Pielou Evenness**	0.03	0.629	0.13	0.358	0.06	0.662	0.13	0.356	−0.35	0.011
**Faith PD**	−0.02	0.831	0.09	0.558	−0.10	0.496	0.01	0.938	−0.33	0.017

WD = Western Diet; Flex = Flexitarian; VG = Vegetarian; VN = Vegan; Faith PD = Faiths’s phylogenetic diversity. Significance levels: *p* < 0.05, significant; *p* < 0.01, highly significant.

**Table 9:** Pearson correlation between fiber compounds (g/day) and alpha diversity in diet types.

		Overall (*n* = 205)		WD (*n* = 50)		Flex (*n* = 52)		VG (*n* = 51)		VN (*n* = 52)	
	Alpha Diversity	*r*	*p*-Value	*r*	*p*-Value	*r*	*p*-Value	*r*	*p*-Value	*r*	*p*-Value
**Fiber non-soluble**	**Shannon Entropy**	−0.09	0.213	0.21	0.139	0.03	0.812	0.13	0.361	−0.33	0.014
	**Pielou Evenness**	−0.06	0.416	0.30	0.037	0.12	0.414	0.11	0.460	−0.33	0.016
	**Faith PD**	−0.09	0.208	0.16	0.258	−0.08	0.587	0.06	0.697	−0.21	0.137
**Fiber soluble**	**Shannon Entropy**	0.07	0.294	0.06	0.707	−0.01	0.970	0.11	0.465	0.17	0.226
	**Pielou Evenness**	0.14	0.045	0.27	0.060	0.02	0.902	0.16	0.277	0.13	0.369
	**Faith PD**	0.05	0.483	−0.04	0.787	−0.01	0.961	−0.02	0.918	0.26	0.060
**Cellulose**	**Shannon Entropy**	−0.10	0.169	0.15	0.146	0.09	0.514	0.12	0.411	−0.30	0.030
	**Pielou Evenness**	−0.06	0.427	0.24	0.100	0.18	0.205	0.17	0.244	−0.31	0.021
	**Faith PD**	−0.01	0.155	0.06	0.668	−0.03	0.834	0.003	0.990	−0.15	0.268
**Starch**	**Shannon Entropy**	−0.12	0.099	0.29	0.038	−0.26	0.067	0.11	0.455	−0.50	0.000
	**Pielou Evenness**	−0.11	0.119	0.19	0.177	−0.16	0.247	0.04	0.784	−0.40	0.003
	**Faith PD**	−0.02	0.740	0.42	0.002	−0.21	0.146	0.09	0.522	−0.30	0.030

WD = Western Diet; Flex = Flexitarian; VG = Vegetarian; VN = Vegan; Faith PD = Faiths’s phylogenetic diversity. Significance levels: *p* < 0.05, significant; *p* < 0.01, highly significant.

**Table 10:** Pearson correlations between mono-, di-, and oligosaccharides and alpha diversity in diet types.

		Overall (*n* = 205)		WD (*n* = 50)		Flex (*n* = 52)		VG (*n* = 51)		VN (*n* = 52)	
	Alpha Diversity	*r*	*p*-Value	*r*	*p*-Value	*r*	*p*-Value	*r*	*p*-Value	*r*	*p*-Value
**Mono-saccharides**	**Shannon Entropy**	0.08	0.275	0.36	0.014	0.28	0.049	0.18	0.208	−0.20	0.150
	**Pielou Evenness**	0.03	0.637	0.34	0.023	0.37	0.007	0.03	0.823	−0.26	0.062
	**Faith PD**	0.06	0.382	0.28	0.063	0.09	0.521	0.32	0.022	−0.12	0.391
**Di-saccharides**	**Shannon Entropy**	0.10	0.146	0.17	0.278	0.13	0.368	0.14	0.330	−0.35	0.010
	**Pielou Evenness**	0.08	0.239	0.12	0.427	0.18	0.193	0.13	0.369	−0.26	0.061
	**Faith PD**	0.08	0.232	0.20	0.182	0.05	0.706	0.13	0.370	−0.32	0.018
**Oligo-saccharides (non-absorbable)**	**Shannon Entropy**	−0.16	0.020	−0.22	0.133	−0.04	0.776	−0.14	0.333	−0.13	0.343
	**Pielou Evenness**	−0.05	0.475	−0.23	0.105	0.10	0.473	0.06	0.683	−0.05	0.718
	**Faith PD**	−0.16	0.026	−0.17	0.239	−0.21	0.136	−0.24	0.088	0.03	0.825
**Oligo-saccharides (absorbable)**	**Shannon Entropy**	−6 × 10^−4^	0.993	0.10	0.476	−0.14	0.328	0.08	0.601	−0.17	0.220
	**Pielou Evenness**	−0.06	0.362	0.08	0.597	−0.08	0.592	0.20	0.158	−0.003	0.982
	**Faith PD**	−0.02	0.796	0.17	0.235	−0.12	0.407	−0.06	0.660	−0.18	0.189

WD = Western Diet; Flex = Flexitarian; VG = Vegetarian; VN = Vegan; Faith PD = Faiths‘s phylogenetic diversity. Significance levels: *p* < 0.05, significant; *p* < 0.01, highly significant.

## Data Availability

The data sets generated and analyzed during the current study are available from the corresponding author on reasonable request. The data are not publicly available due to the ongoing evaluation of the data sets (intervention period, follow-up) by the study team.

## Supplementary Materials

The following supporting information can be downloaded at https://www.mdpi.com/article/10.3390/nu15081914/s1, Figure S1: Work flow of the statistical assessment; Figure S2: Relative abundance of the 10 top most genera split by diet form. Remaining entries were merged as “Others”; Supplementary Table S1: Relative abundancies of the four diets test and their sum used for the rankings.
