# Supplementary material for: Role of Dietary Fiber and Energy Intake on Gut Microbiome in Vegans, Vegetarians, and Flexitarians in Comparison to Omnivores—Insights from the Nutritional Evaluation (NuEva) Study
Source: Nutrients. 2023 Apr 15;15(8):1914. doi: 10.3390/nu15081914 (PMC10146654; doi:10.3390/nu15081914)
Supplement: Supplementary file 1 [file nutrients-15-01914-s001.zip › nutrients-2324990-supplementary.pdf]

Supplement Table S1: Relative abundancies of the four diets test and their sum used for the rankings.

%	WD	Flex	VG	VN	Total
Bacteroides	12.8	16.1	18	18.9	65.8
Blautia	8.9	8.4	7.3	7.9	32.5
Faecalibacterium	7.0	7.1	6.9	7.8	28.8
Prevotella	7.2	6.5	4.7	4.1	22.5
Agathobacter	3.0	3.3	2.4	3.8	12.5
Bifidobacterium	2.1	2.9	3.3	3.1	11.4
Anaerostipes	2.8	3.0	2.5	2.9	11.2
Subdoligranulum	2.8	2.7	2.7	2.7	10.9
Alistipes	2.2	2.3	3.0	2.5	10.0
Roseburia	2.0	2.0	2.5	3.3	9.8
Eubacterium_hallii_group	2.4	2.2	2.3	2.0	8.9
Fusicatenibacter	2.2	2.3	2.0	2.1	8.6
Ruminococcus	2.1	1.8	2.1	1.9	7.9
UCG_002	1.9	1.8	2	1.4	7.1
Parabacteroides	1.5	1.7	1.8	2.0	7.0
Not_Assigned	1.7	1.7	1.5	2.0	6.9
Coprococcus	1.8	1.4	1.7	1.5	6.4
Christensenellaceae_R_7_group	1.4	1.7	1.7	1.6	6.4
Akkermansia	1.5	1.4	2.3	1.2	6.4
CAG_352	1.9	1.4	1.1	1.3	5.7

Work flow

Analyses across all diet types (WD, Flex, VG, VN) and between diet types (Flex, VG, VN) compared to the reference diet (WD)

Dietary intake

Dietary intake of carbohydrates, fibers and energy

Microbiome composition

alpha diversity

> Pearson correlation between carbohydrate intake (g/day) / fiber intake (g/day) / energy intake (kcal/day) / fiber compounds (g/day) /mono-, di-, and oligosaccharides and alpha diversity in diet types

beta diversity

> beta diversity metrics comparison against WD / reference diet split between high fiber and low fiber content groups

Firmicutes/Bacteriodetes-Ratio distribution for each diet

LefSe analysis to identify biomarkers for the studied diets

Correlation analysis between identified biomarkers for the extreme ends of the spectrum (WD and VN) and cardiovascular risk factors

Supplement Figure S1: The work flow of the statistical assessment

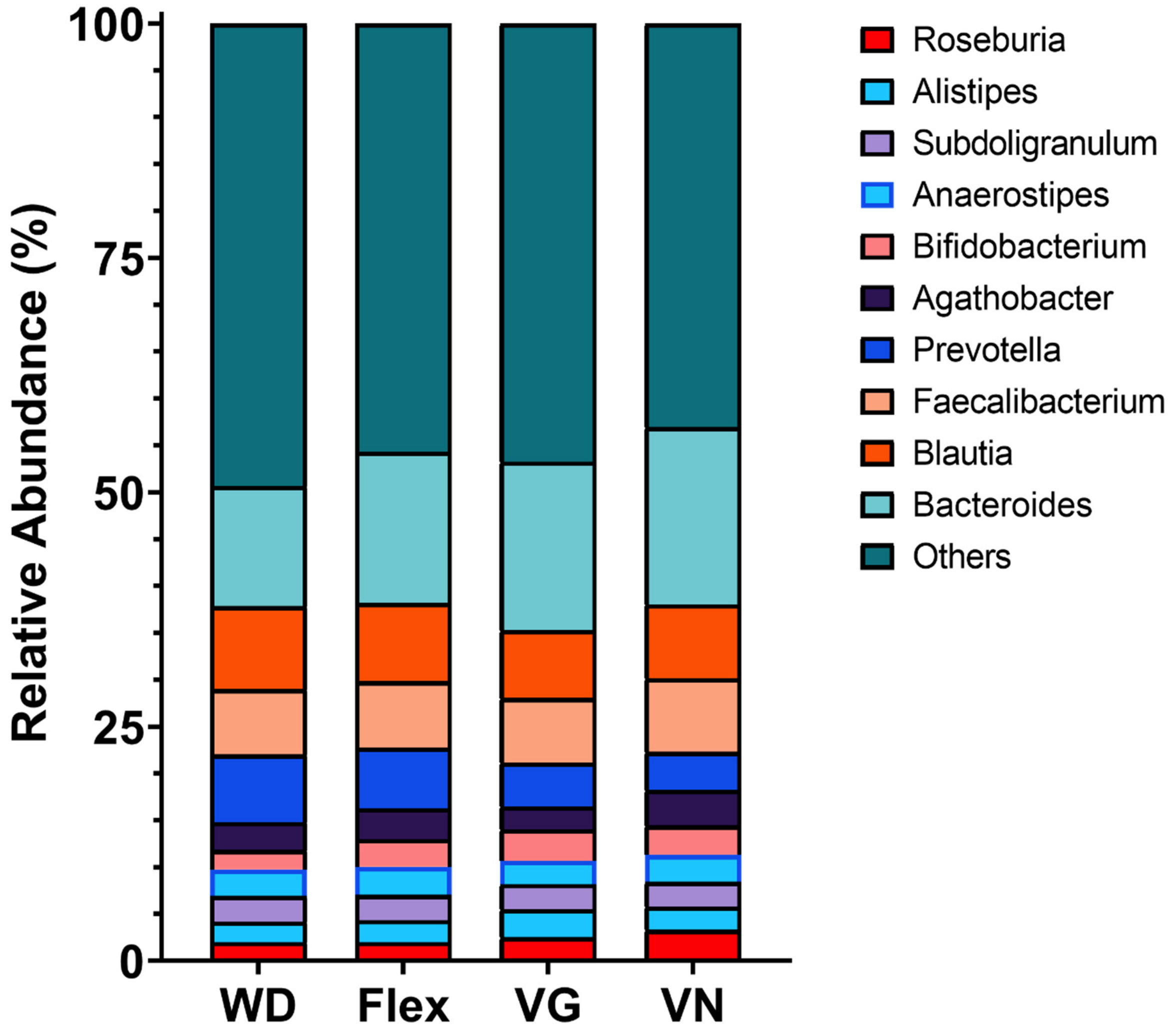

Supplement Figure S2: Relative abundance of the 10 top most genera split by diet form. Remaining entries were merged as “Others”
